# COVID-19 Pneumonia on Post-Operative Day 2 after Esophagectomy: Performing Esophago-Gastric Junction Cancer Surgery during the SARS-Cov-2 Second Wave

**DOI:** 10.3390/curroncol28020128

**Published:** 2021-03-27

**Authors:** Kamil Nurczyk, Chia-En Chan, Norbert Nowak, Tomasz Skoczylas

**Affiliations:** 2nd Department of General Surgery, Medical University of Lublin, ul. Staszica 16, 20-400 Lublin, Poland; chiaen.chan@gmail.com (C.-E.C.); nnowak@spsk1.lublin.pl (N.N.); tskoczylas@spsk1.lublin.pl (T.S.)

**Keywords:** gastro-esophageal junction cancer, esophagectomy, COVID-19, pneumonia

## Abstract

The coronavirus disease 2019 (COVID-19) pandemic has had a substantial impact on the provision of medical healthcare. Due to an increased risk of severe acute respiratory syndrome coronavirus 2 (SARS-Cov-2) transmission, elective surgical treatment has been suspended in many centers. The effects of COVID-19 in the early post-operative period after esophagectomy remains unknown. In this report, we present three cases of patients diagnosed with esophago-gastric junction cancer who were scheduled for elective esophagectomy with a curative intention during second wave of COVID-19 pandemic in a single high-volume tertiary center. Despite all available safety measures, one of the patients developed COVID-19 pneumonia on post-operative day two, leading to an impaired respiratory function and increased pleural fluid collection from the chest tube, resulting in a prolonged time of hospital stay. Finding a good balance between the COVID-19-related perioperative risks and consequences of delaying surgical treatment in patients diagnosed with esophago-gastric cancer is a challenge. In order to achieve the best possible outcome, care must be taken to ensure availability of necessary treatment options and to reduce the risk of SARS-Cov-2 transmission perioperatively.

## 1. Introduction

The coronavirus disease 2019 (COVID-19) has been labeled as a pandemic by the World Health Organization (WHO) since 11 March 2020 [1] and has had a substantial impact on the provision of medical healthcare worldwide. Concerns regarding the increased risk of contracting the virus resulted in periodical suspension of elective surgeries in many centers. The correlation between COVID-19 infection in the post-operative period and surgical outcome has elicited varying perspectives among experts [2,3,4,5]. However, it may be reasonable to consider that cancer patients are associated with poorer outcomes from COVID-19 [6]. Little evidence is available on COVID-19′s effects on thoracic surgeries, but it does confer greater mortality and comorbidities [7].

Surgical approaches combined with neoadjuvant chemotherapy are considered the main treatment option for esophageal cancer [8]. The objective of the Ivor Lewis esophagectomy in cancer patients is to remove the tumor by resecting the lower part of esophagus and proximal part of stomach. The procedure also involves lymphadenectomy and reconstruction with esophago-gastric anastomosis in the posterior mediastinum. The operation requires access to the abdominal cavity and chest, affecting the extent of the surgical injury.

Patients following esophagectomy are at risk of respiratory complications (mainly pneumonia), conduit necrosis, anastomotic leak, and cardiac disorders, such as atrial fibrillation [7,9,10,11]. Connecting an essential feature of transthoracic esophagectomy, the association with increased risk of pulmonary complications, with the scenario of severe acute respiratory syndrome coronavirus 2 (SARS-Cov-2)-induced pneumonia in early post-operative days (PODs) stresses an exceptional concern on the prognosis of esophago-gastric junction (EGJ) cancer patients. At this point, data on the effects of COVID-19 on the post-operative period after esophagectomy are rudimentary [12]. Therefore, we present a case of COVID-19 pneumonia on POD 2, following Ivor Lewis esophagectomy for EGJ cancer.

## 2. Materials and Methods

This report presents all three patients diagnosed with EGJ cancer who underwent elective surgery with the curative intention in the 2nd Department of General Surgery, Medical University of Lublin, during the second wave of SARS-Cov-2 pandemic in Poland, between November 1 and November 30, 2020. Written informed consent has been obtained from the patients to publish this paper. The study was conducted according to the guidelines of the Declaration of Helsinki and approved by the Ethics Committee of the Medical University of Lublin (KE-0254/297/2016).

## 3. Case Reports

### 3.1. Patient 1

An otherwise healthy 37-year-old man who reported increasing symptoms of dysphagia over 6 weeks underwent endoscopic evaluation. Esophagogastroduodenoscopy (EGD) revealed a tumor in the EGJ 36 cm to 41 cm from the incisors. The endoscopic tumor biopsy indicated a G2 Lauren intestinal-type adenocarcinoma. Computed tomography (CT) scanning showed regional lymph nodes involvement with no distant metastases. The pre-treatment clinical diagnosis based on TNM staging [13] was cT4aN2M0.

Neoadjuvant chemoradiotherapy (NACRT) according to the CROSS protocol (carboplatin/paclitaxel) was initiated. A total dose of 41.8 Gy was administered. Restaging based on CT scan and EGD was performed after completing NACRT. Tumor response was not observed and evaluated as stable disease (SD) according to RECIST criteria. The post-NACRT clinical TNM stage was described as cT4aN2M0.

Consequently, the patient was scheduled for elective Ivor Lewis esophagectomy 8 weeks after the completion of NACRT. According to the local epidemiological guidelines, he was encouraged to self-isolate for 10 days prior to admission. A routine nasopharyngeal swab RT-PCR test for SARS-Cov-2 was performed with a negative result upon admission to the Department of Surgery, and the patient did not report any symptoms of infection.

The patient underwent esophagectomy with two-field lymphadenectomy, gastric pull-up reconstruction, and linear stapler esophago-gastric anastomosis at the level of the azygos vein arch, plus feeding jejunostomy placed 40 cm below the ligament of Treitz. Surgery lasted 400 min, and blood loss was 450 cc. No intraoperative complications were reported.

At the day of surgery, the patient remained stable and was extubated. The post-operative course was uneventful. No signs of infection were reported, and control chest x-rays did not reveal any pleural infiltration. After removing the drain from the left pleural cavity, introducing oral intake, and removing the jejunostomy tube, the patient was discharged home on POD 8 and scheduled for chemotherapy.

### 3.2. Patient 2

A 69-year-old man reported symptoms of dysphagia and weight loss of 6 kg over several weeks. Other comorbidities included hypertension, coronary artery disease, diabetes mellitus type 2, and gout arthritis. He underwent EGD, revealing a tumor in the EGJ 40 cm to 46 cm from the incisors. The tumor biopsy indicated a G2 Lauren mixed-type adenocarcinoma. Computed tomography (CT) scanning revealed regional lymph node involvement with no distant metastases. The pre-treatment clinical diagnosis was cT4aN2M0.

CROSS NACRT was initiated. A total dose of 41.8 Gy was administered. Restaging based on a CT scan and EGD was performed after completing NACRT. The tumor response was evaluated as SD. The patient was scheduled for an elective Ivor Lewis Esophagectomy 8 weeks after the completion of NACRT. After a 10-day self-isolation, a routine nasopharyngeal swab RT-PCR test result for SARS-Cov-2 was negative on admission to the hospital.

Although the patient was qualified for surgery with a therapeutic intention, the intraoperative evaluation of the tumor showed that it was unresectable due to infiltration of the diaphragm, liver, and aorta, which were not previously detected on CT. Therefore, surgery was limited to a G-tube feeding gastrostomy. The procedure lasted 100 min, and estimated blood loss was less than 50 mL. No complications were reported.

The post-operative course was uneventful. After introducing gastrostomy feeding on POD 1, achieving good pain control, and initial recovery, the patient was discharged home on POD 4 and scheduled for chemotherapy.

### 3.3. Patient 3

A 68-year-old man who presented dysphagia and weight loss of 4 kg over 1 month underwent evaluation for suspected esophageal carcinoma. Other comorbidities included hypertension and rheumatoid arthritis. EGD revealed a tumor in the EGJ 36 cm to 44 cm from the incisors (EGJ at the level of 42 cm). The tumor biopsy indicated a G2 Lauren mixed-type adenocarcinoma. CT scanning indicated regional lymph node involvement. No distant metastases were found. The pre-treatment clinical diagnosis was cT4aN2M0.

NCRT according to the CROSS protocol was initiated. A total dose of 45 Gy was administered. Restaging based on a CT scan and EGD was performed after completing NACRT. The tumor response was limited and the post-NACRT clinical TNM stage was described as cT4aN2M0.

Consequently, the patient was scheduled for elective surgery 8 weeks after the completion of NACRT. The patient self-isolated for 10 days prior to admission. A routine nasopharyngeal swab RT-PCR test for SARS-Cov-2 was performed with a negative result upon admission to the Department of Surgery, and the patient did not report any symptoms of infection.

The patient underwent a laparotomy and a right thoracotomy Ivor Lewis esophagectomy with two-field lymphadenectomy and gastric pull-up reconstruction with linear stapler esophago-gastric anastomosis at the level of the azygos vein arch. Feeding jejunostomy was placed 40 cm below the ligament of Treitz. The surgery lasted 330 min and blood loss was 350 cc. As a result of an excessive operating field exposition using intercostal retractor during thoracotomy, a fracture of the fifth rib occurred. No other complications were reported.

At the day of surgery, the patient remained stable and was extubated. On POD 1, the patient required increased epidural doses of analgesics due to a high post-operative pain level and several interventions to control high blood pressure (BP) (up to 210/87 mmHg). In addition, an episode of atrial fibrillation (AF) was reported but was successfully controlled pharmacologically. A bedside chest x-ray revealed a small left-sided pleural effusion. One thousand one hundred mL of serous fluid was drained from a chest tube located in the right pleural cavity. Fever was not reported. Empiric antimicrobial treatment was administered (Meropenem 1000 mg i.v., every 12 h).

**Figure 1:**
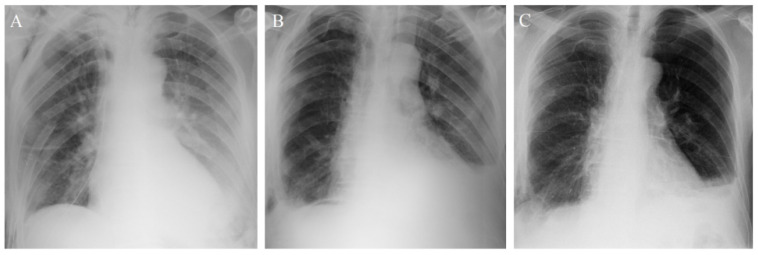
Chest x-rays from post-operative day 2 (**A**), post-operative day 7 (**B**), post-operative day 18 (**C**).

On POD 2, despite good pain control, the patient required antihypertensive treatment and experienced another episode of AF but with good response to pharmacological treatment. At the end of POD 2, the patient reported dyspnea, SPO2 dropped below 80% and respiratory rate (RR) was 20 breaths/min. He required an increase in oxygen concentration use and a change from a conventional O2 mask to Venturi mask oxygen therapy. The ECG and troponine levels were within normal ranges. A bedside chest X-ray revealed interstitial infiltration in the lower zones of the lungs (Figure 1). Fever was not reported. At this point the oropharyngeal swab test for the SARS-Cov-2 antigen was performed with a positive result, and the COVID-19 infection was confirmed by using RT-PCR assay.

On POD 3, the patient’s vital signs were stable and a SPO2 of 95% was achieved using a Venturi mask. On POD 4 and 5, the patient tolerated low concentration oxygenation with a normal O2 mask and did not present symptoms of dyspnea. However, the amount of fluid drained from right pleural cavity remained relatively high. The NG tube was removed on POD 4, and clear liquids were introduced on POD 5.

On POD 6, the patient’s ventilation improved significantly. A bedside chest x-ray revealed no progression in interstitial infiltration in the lower zone of the left lung and remaining signs of left pleural effusion. The fluid collected from the chest tube dropped to 200 mL. A full liquid diet was introduced. On POD 7, the chest tube was removed.

A bedside chest x-ray examination on POD 8 showed no progression in radiological signs of pneumonia. During the second week after the surgery, the patient’s vital signs remained stable. The pain control was optimal, and intensive physical therapy was continued. The patient periodically required low-concentration oxygen therapy. Oral nutrition was used in combination with enteral jejunostomy feeding. On POD 9, the jejunostomy tube was removed. On POD 10, the patient was transferred from surgical ICU to the Infectious Disease Department and continued therapy.

A control chest x-ray on POD 18 showed reduction in bilateral lower zones’ opacities and pleural effusions. The patient was discharged on POD 19, and treatment was continued at home. Later he was scheduled for chemotherapy without any delay. Data regarding the post-operative course are summarized in Table 1. Serie of chest-x rays are shown in Figure 1.

## 4. Discussion

We all hope that COVID-19 vaccines will eventually help contain the pandemic. However, it is difficult to predict the extent of vaccination in the entire population, as well as the pace of the vaccination process and its impact on reducing the number of infections. In addition, the situation has been complicated by new strains of the virus. Therefore, probably in the next months, self-isolation of patients undergoing elective surgery and extensive screening for asymptomatic infection will remain the only way to increase safety [14].

Unfortunately, even the best preventive measures cannot guarantee avoidance of hospital admission and operating on false-negative patients. Despite the threat, some procedures cannot be delayed. Since the COVID-19 outbreak, the strategy for esophageal cancer treatment during the pandemic has been disputed [15]. The major concern is the impact on treatment delay, resulting in disease progression [16,17]. According to actual guidelines, it is suggested that esophagectomy in patients with esophageal cancer should be performed within 6–8 weeks after the completion of neoadjuvant treatment [18,19,20].

Balancing between the risks of COVID-19-related perioperative complications and consequences of delaying surgical treatment is a challenge in EGJ cancer patients. However, available data indicate that EGJ cancer surgery can be successfully carried out without compromising patient outcomes [21].

Due to the small number of cases, the data we presented do not allow for estimation of the risk of developing COVID-19 in the perioperative period after esophagectomy during the time of a pandemic wave. These limitations also prevent us from drawing far-reaching conclusions about the results for these patients. However, it should be assumed that the risk is present and has a major impact on the post-operative course. Comparing the clinical course of the presented patient 3, several observations can be made: (1) COVID-19 pneumonia symptoms may be difficult to distinguish from early anastomotic complications. This concern resulted in a delay in the time of NG tube removal and oral intake introduction; (2) increased pleural fluid collection also delayed the chest tube removal; (3) impaired respiratory function hindered proper post-operative physical therapy and recovery; (4) in consequence, the length of hospital stay was prolonged compared to non-complicated cases.

An additional problem to overcome was the increased risk of secondary transmission of SARS-Cov-2 in the hospital setting due to the number of personnel necessary for multidisciplinary treatment. We did not report any in-hospital transmission to other patients or personnel. However, this may prove to be particularly important in resource-limited hospital settings, where preserving the resources, such as personal protective equipment, hospital space, and personnel time, may be necessary to cover acute and urgent cases.

## 5. Conclusions

It is a struggle to dictate whether high risk patients should undergo such extensive surgery knowing that COVID-19 can complicate the perioperative course of patients undergoing esophagectomy by prolonging the recovery process and hospital stay.

In this case series presented, our study established that COVID-19 does have a significant impact on the post-operative recovery course. Although situated in a desperate time, EGJ cancer surgery is still feasible, provided that protective precautions are made and certain guidelines are met.

Increased risk of complications and mortality during a worldwide healthcare crisis requires individualized assessment of potential risks and benefits for performing elective esophagectomy to improve patient outcomes and minimize the burden on the health care system.

## Figures and Tables

**Table 1:** Time course in terms of clinical laboratory results, symptoms, and interventions. RT-PCR, Reverse transcription polymerase chain reaction; WBC, white blood count; CRP, C-reactive protein serum level; PCT, procalcytonin serum level. The colors indicate the presence of a given parameter’.

Post-Operative Day	1	2	3	4	5	6	7	8	9	10	11	12	13	14	15	16	17	18	19
SARS-Cov-2 antigen swab test		(+)																	(−)
RT-PCR of SARS-Cov-2 swab test		(+)									(+)								(+)
WBC (K/uL)	5.11	6.08	5.64	5.26		4.20		3.31			2.34				2.49				2.87
CRP (mg/L)	167.2	196.8	126.3	86.0		42.7		17.4			16.5				36.5				36.7
PCT (ng/mL)	0.42	2.95	1.37	0.57		0.24													
Chest tube collection (mL/24 h)	1100	850	700	900	450	200	50												
Hypertension (systolic BP > 140 mmHg)																			
Atrial fibrillation																			
Dyspnea																			
Antimicrobial tx: Meropenem		1 g i.v./12 h																	
Jejunostomy enteral feeding (mL/h)	15	30	45	60	60	60	60	60	60										
Oral intake																			

## Data Availability

The data presented in this study are available on request from the corresponding author. The data are not publicly available due to terms of patient consent.
